# The interaction between intratumoral bacteria and metabolic distortion in hepatocellular carcinoma

**DOI:** 10.1186/s12967-024-05036-7

**Published:** 2024-03-04

**Authors:** Chen Xue, Xinyu Gu, Qingmiao Shi, Xiao Ma, Junjun Jia, Yuanshuai Su, Zhengyi Bao, Juan Lu, Lanjuan Li

**Affiliations:** 1 State Key Laboratory for Diagnosis and Treatment of Infectious Diseases, National Clinical Research Center for Infectious Diseases, National Medical Center for Infectious Diseases, Collaborative Innovation Center for Diagnosis and Treatment of Infectious Diseases, The First Affiliated Hospital, Zhejiang University School of Medicine, Hangzhou, Zhejiang China; 2 Department of Oncology, College of Clinical Medicine, The First Affiliated Hospital, Henan University of Science and Technology, Luoyang, Henan China; 3 Division of Hepatobiliary and Pancreatic Surgery, Department of Surgery, The First Affiliated Hospital, Zhejiang University School of Medicine, Hangzhou, Zhejiang China

**Keywords:** Hepatocellular carcinoma, Intratumor bacteria, Metabolites, Biomarker, Therapeutic targets

## Abstract

**Background:**

Intratumoral bacteria might play essential roles in tumorigenesis in different cancer types. However, its features and potential roles in hepatocellular carcinoma (HCC) are largely unknown.

**Methods:**

In this study, we assessed bacterial RNA by 16S rRNA fluorescence in situ hybridization and detected bacterial lipopolysaccharide (LPS) via immunohistochemistry. Hepa1-6 cells were used to establish orthotopic HCC models in mice. 2bRAD sequencing for microbiome was performed to determine the intratumoral bacterial characteristics, and liquid chromatography-mass spectrometry was conducted to explore the metabolic profile. The potential association between different intratumoral microbiota and metabolites were evaluated.

**Results:**

We detected bacterial 16S rRNA and LPS in HCC tissues from the patients with HCC. In HCC mouse model, we found that the intratumor bacteria in HCC tissues were significantly different to adjacent nontumor tissues. Furthermore, we observed different metabolites in HCC tissues and adjacent nontumor tissues, such as N-acetyl-D-glucosamine and a-lactose. Our results showed that several bacteria were significantly associated with metabolites, such as *Pseudomonas koreensis*, which was positively correlated with N-acetyl-D-glucosamine and negatively correlated with citrulline.

**Conclusions:**

This study confirmed the close association between different bacteria and metabolites, which might provide novel opportunities for developing new biomarkers and therapeutic targets for HCC.

**Supplementary Information:**

The online version contains supplementary material available at 10.1186/s12967-024-05036-7.

## Background

Primary liver cancer is the fourth leading cause of cancer-related mortality worldwide, of which hepatocellular carcinoma (HCC) accounts for 80–90% [1, 2]. About 800,000 people worldwide die each year from HCC [3], which is often caused by viral hepatitis B or C, persistent alcohol abuse and nonalcoholic fatty liver disease. Given the global increase in obesity and type 2 diabetes, metabolic syndrome-related nonalcoholic fatty liver disease has become an increasingly risk factor for HCC [4, 5]. As the early symptoms and features of HCC are not typical, more than 80% of patients with HCC cannot receive curative treatment [6]. Therefore, the early diagnosis and treatment of HCC still require further exploration.

Previous studies have found that approximately 15% of cancers are attributable to microbial infections [7]. The intestinal microbiome is the most common factor dominating tumor initiation, development, and therapeutic efficacy [8–11]. Over the years, accumulating evidence has proved that polymorphic intratumoral microbiomes enable cancer hallmark capabilities and have been recognized as emerging mechanisms of tumorigenesis and progression [12–14]. Various tumors, including HCC, were once considered sterile tissues. However, advances in sequencing technology have led to the identification and characterization of the microbial composition in tumor tissues. The classical technologies of 16S rRNA, 18S rRNA and whole genome assessments have been extensively applied to obtain the taxonomic profile of the microbiome. A new method, 2bRAD sequencing for microbiome (2bRAD-M), overcomes the challenge of low microbial biomass or severe DNA degradation in the detected samples [15]. 2bRAD-M uses the type IIB restriction enzyme to perform qualitative and relative quantitative microbial analysis of the unique tags obtained after enzymatic digestion of the microbial genome. This method allows for the accurate generation of species-level taxonomic signatures for low microbial biomasses in tumor tissues and normal tissues [16, 17].

Microbial metabolism is the essential characteristic and function of microbes, and most of their effects on the host are related to microbial metabolism. Previous researches have extensively explored the relationship between gut microbiota and metabolism through multi-omics integration analysis [18–20], but clinical data on intratumoral bacteria and the relationship between the intratumoral microbiome and metabolome have not been widely studied.

Unlike gut microbiomes, tumor tissues are often of low biomass, and therefore we assessed bacterial 16S rRNA using the fluorescence in situ hybridization (FISH) method to verify the presence of bacteria in tumor and normal tissues of HCC patients. In addition, we constructed a mouse liver cancer model in situ and analyzed the intratumoral microbial community (2bRAD-M, n = 24) and metabolome (LC–MS, n = 24) from tumor tissues and normal liver tissues obtained from the HCC mouse model. The characterization of tumor microbiome and metabolome might provide new opportunities for developing novel biomarkers and therapeutic targets.

## Materials and methods

### Histology analysis

A total of 3 pairs of HCC tissues and adjacent normal tissues were collected from the First Affiliated Hospital, Zhejiang University School of Medicine. The study was conducted in accordance with the Declaration of Helsinki, and was approved by the Ethics Committee of the First Affiliated Hospital, Zhejiang University School of Medicine (No. IIT20210168B-R1).

HCC tissues and adjacent liver tissues from HCC patients were paraffin-embedded and sectioned at a 40 µm thickness. Hematoxylin and eosin (H&E) stained the cytoplasm and nucleus with contrasting colors to identify cellular components. For immunohistochemistry (IHC) staining, HCC sections were dewaxed with xylene and hydrated with absolute ethanol. The tissue sections were immersed in citric acid antigen retrieval buffer. Then, the samples were heated for 8 min to boiling, taken off the heat for 8 min, and then returned to a medium–low heat for 7 min. The slices were washed 3 times in phosphate buffer solution (PBS) (pH 7.4) with shaking on a decolorizing shaker for 5 min each time. Then, the tissue sections were immersed in 0.3% H_2_O_2_-methanol for 25 min, washed with PBS, and probed with the anti-*Salmonella typhimurium* lipopolysaccharide (LPS) antibody (Abcam, Cambridge, UK) and a rabbit polyclonal anti-Ki67 antibody (Abcam, Cambridge, UK) at 4 °C overnight. On the second day, the slices were washed with PBS, and horseradish peroxidase-conjugated goat anti-rabbit secondary antibody was added and incubated at room temperature for 1 h. Liver sections were scanned with panoramic MIDI (3DHISTECH, Budapest, Hungary).

### 16S rRNA fluorescence in situ hybridization (FISH)

FISH was performed to detect bacteria in HCC tissues. The probe used was the universal 16S rRNA probe EUB338 (5’-CY3-GCTGCCTCCCGTAGGAGT-3’), which is used to specifically bind to bacterial 16S rRNA. After precipitating the probes, sample slides were processed with prehybridization, hybridization, and posthybridization washes and DNA counterstaining, and the procedure was performed according to published studies [13, 21].

### Cell culture

The murine HCC cell line Hepa1-6 was obtained from American Type Culture Collection (ATCC, USA). Hepa1-6 cells were cultured in high glucose Dulbecco’s modified Eagle’s medium (Sigma-Aldrich, USA) supplemented with 10% fetal bovine serum (Sigma-Aldrich, USA) and 1% penicillin- streptomycin (Thermo Fisher Scientific, USA). Prior to experiments, cells were maintained in an incubator at 37 °C in a 5% CO_2_ atmosphere.

### Orthotopic HCC mouse model

The animal experiment was approved by the Animal Experimental Ethics Committee of The First Affiliated Hospital, Zhejiang University School of Medicine (project number 20221072). Six-week-old WT C57BL/6 J male mice (n = 12) were obtained from the Experimental Animal Center of Zhejiang Academy of Medical Sciences. All animals were housed under specific pathogen-free conditions at a constant temperature (22 ± 2 °C) with a 12-h daylight/darkness cycle, and they had free access to standard rodent feed and tap water until the end of the experiment.

After a week of adaptive feeding, the mice were anesthetized and placed on a laboratory table in the supine position. After disinfecting the abdomen of the mice, sterilized small forceps and ophthalmic scissors were used to open the mouse abdomen along the middle line of the abdomen to avoid unnecessary bleeding. The left lobe of the liver was removed using sterile swabs. A total of 3 × 10^5^ Hepa1-6 cells in 10 µl Corning Matrigel (Matrigel:PBS = 1:4) were injected into the left lobe of the liver, which was then placed back into the abdominal cavity. The surgical site was sutured and disinfected with iodophor. Continuous monitoring and care were given to mice after surgery. After 2 weeks, the HCC tissues and paired adjacent liver tissues were harvested from mice for further analysis. During the processes of model construction, liver samples handling, transportation and sequencing, strict aseptic procedures were followed to eliminate potential bacterial contamination.

### 2bRAD sequencing for microbiome (2bRAD-M)

2bRAD-M is a microbial diversity analysis technique based on 2b-RAD technology [22], which performs qualitative and relative quantitative analysis of microorganisms by unique tags obtained after enzymatic cleavage of microbial genomes by type IIB restriction enzymes. A database containing unique tags of each microorganism (2b-Tag-DB) was used for qualitative analysis [15, 22]; that is, all microbial species that had unique tags were screened. The 2b-Tag-DB was established again for the quantitative microorganisms, and the relative quantitative analysis was carried out; that is, the microbial species obtained in the previous step were subsequently screened, and the abundance was estimated according to the distribution of unique tags.

### Bioinformatics analysis of the microbiome

Microbial genomes were electronically cleaved using the BcgI restriction enzyme to extract raw reads. The clean reads for each sample were searched separately in the 2bRAD-M database (http://github.com/shihuang047/2bRAD-M) to obtain microbial annotation information for that sample. The clean reads of each sample were retrieved in the new database using a secondary library built using the genomes of the microorganisms that might be present, and the relative abundance of each microorganism in the sample was calculated using the special formula [15, 17]. Finally, the samples were annotated and summarized at different classification levels, such as phylum, genus and species. The R package Vegan v2.5.7 was used to perform PCoA ordination.

### Metabolomics analysis

Liquid chromatography-mass spectrometry (LC–MS) technology [23] was used in this nontargeted metabolomics research [24, 25]. The experimental process mainly included metabolite extraction, LC–MS detection and data analysis. Samples were stored at −80 °C after collection until analysis. In brief, HCC tissues and liver tissues were mixed with 300 μL of precooled acetonitrile and 200 mg of ceramic beads. Next, the mix was homogenized and centrifuged (4 °C, 12,000 rpm) for 10 min. The supernatant was centrifuged (4 °C, 12 000 rpm, 10 min) again and then filtered using 0.22 μm syringe filters before analysis. The QExactiveTM HF mass spectrometer was operated in positive and negative polarity mode with a spray voltage of 3.5 kV, sheath gas flow rate of 35 psi, capillary temp of 320 °C, and aux gas flow rate of 10 L/min [26, 27].

### Bioinformatics analysis of metabolomics

The raw data were first analyzed using Compound Discoverer 3.1 (CD3.1) software. The peaks were simply screened, and were aligned according to retention time deviation and mass deviation for different samples to make the identification more accurate. Subsequently, all the peaks were extracted based on the set ppm, signal-to-noise ratio (S/N), additive ions and other information, and the peak area was quantified. Then, spectroscopic processing and database retrieval were conducted to obtain qualitative and quantitative results of metabolites by comparing high-resolution secondary spectral databases mz Cloud and mz Vault and Mass List primary database retrieval, and then quality control was carried out on the data to ensure the accuracy and reliability of data results. Next, the metabolites were subjected to principal component analysis (PCA) and partial least squares discriminant analysis (PLS-DA). Hierarchical clustering (HCA) and metabolite correlation analysis were used to reveal the relationships between samples and metabolites. The KEGG database was used to identify potential biological pathways [28].

### Statistical analysis

Statistical analyses were performed using SPSS (version 26) and the R software (version 4.0). Comparisons between two groups were calculated by Student's t test. In addition, spearman correlation analysis was used to calculate the relationship between two groups based on relative abundance. P < 0.05 was considered statistically significant.

## Results

### Bacterial RNA and lipopolysaccharide (LPS) are present in tumor tissues of HCC patients

**Fig. 1:**
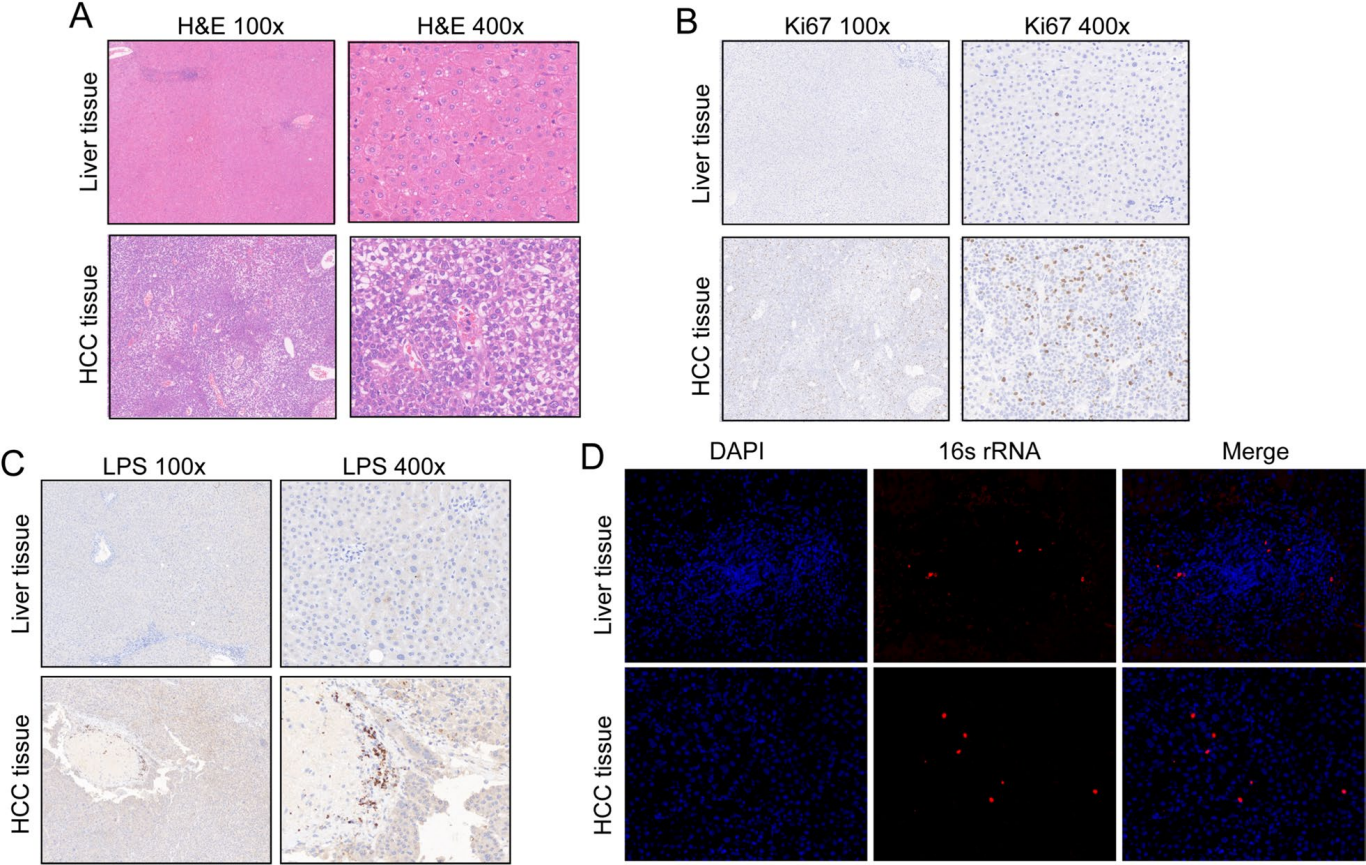
Bacterial components exist in cancer tissues and peritumoral normal tissues of HCC patients. Representative images of the liver tissue and HCC tissue with **A** H&E staining, **B** Ki67 immunohistochemistry staining, **C** LPS immunohistochemistry staining. Images taken at 10 × and 40 × magnification, respectively. **D** Representative confocal micrographs of 16S rRNA FISH in human liver tissue and HCC tissue. Cellular nuclei are labeled in blue, and bacteria in red. Images taken at 40 × magnification. Scale bars = 20 μm

Due to the low biomass of tumor microbiome, verifying the presence of bacteria in tumor samples is challenging. In order to characterize and visualize intratumor bacteria, we performed 16S rRNA fluorescence in situ hybridization (FISH) detection and bacterial outer membrane LPS immunohistochemical (IHC) staining on 3 pairs of human HCC tissues and paracancer normal tissues. H&E staining (Fig. 1A) and proliferative marker Ki67 IHC staining (Fig. 1B) suggested abnormal proliferation of tumor cells in HCC patients. Compared with normal liver tissue, the expression of Ki67 was significantly higher in the nucleus of HCC, indicating that HCC cells were highly malignant. In addition, the bacterial LPS expression was low in normal liver tissue, weakly positive (+ , light brown particles) in the cytoplasm of hepatocellular carcinoma cells, and strongly positive (+ +  + , dark brown particles) in the edge of complete necrosis area of tumor tissue (Fig. 1C). Bacterial 16S rRNA was detected in HCC tissues by FISH (Fig. 1D). The result demonstrated the presence of bacteria inside HCC tissue, and the discrepancy in bacteria between HCC tissues and normal liver might be associated with HCC cell growth.

### Differences in microbial diversity between HCC tissues and normal liver tissues in mouse models

**Fig. 2:**
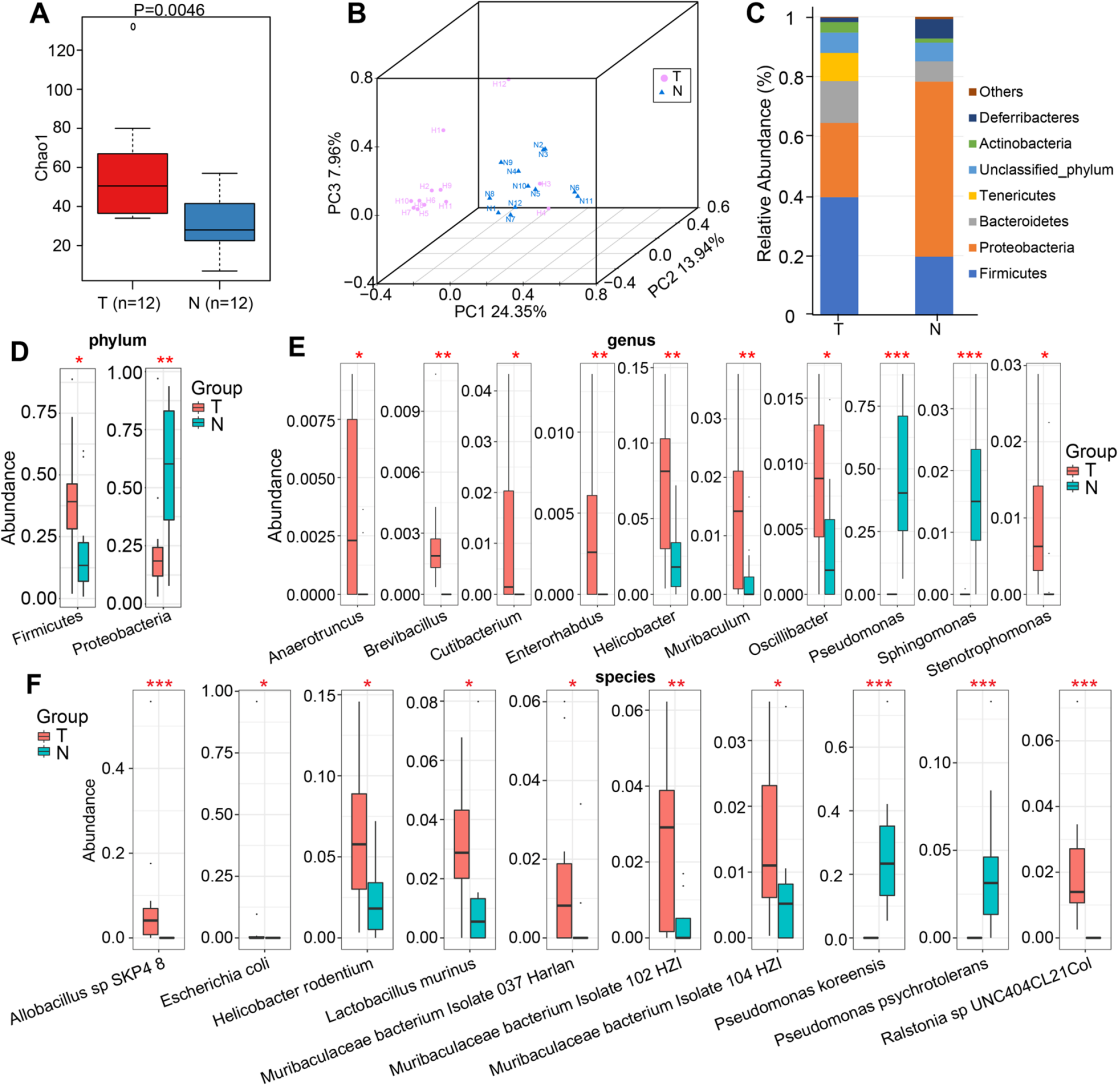
Microbial diversity of HCC tissues and normal liver tissues in mice. **A** Microbial alpha diversity estimated by Chao1 index was significantly increased in HCC tissues compared with normal liver tissues. **B** Beta diversity was calculated using PCoA based on Bray–Curtis distance (*p* = 0.001), respectively. It indicated distinctly different microbiota compositions between HCC and normal liver tissues. **C** Composition of microbiota at phylum level in HCC tissues versus normal liver tissues. Comparison of microbiota at the **D** phylum, **E** genus, and **F** species levels between groups. *PCoA* principal coordinates analysis

We performed 2bRAD-M to compare the microbial community differences between HCC tissue and normal liver tissue from mouse models. Microbial alpha diversity estimated by Chao1 index was significantly increased in HCC tissues compared with normal liver tissues (P = 0.0046) (Fig. 2A). The β-diversity was determined by principal coordinates analysis (PCoA) of unweighted UniFrac PC1-3 to describe the microbiome space of different samples. Based on Bray–Curtis distance (P = 0.001), it showed that the microbial communities in HCC tissues and normal liver tissues were notably separated in the direction of the PC1 axis, which represented that the overall microbial communities were prominently different between HCC tissues and normal liver tissues (Fig. 2B).

### Microbial communities’ structure between HCC tissues and normal liver tissues in mouse models

The microbial communities’ structure of each sample at phylum, genus, and species levels were presented in Additional file 1: Figure S1. At the phylum level, a total of 11 and 10 phyla were identified in the HCC tissues and normal liver tissues, respectively. Overall, Firmicutes was the dominant phylum in the tumor group (39.59%), followed by Proteobacteria (24.91%) and Bacteroidetes (14.00%), while Proteobacteria was the major phylum in the normal liver group (58.66%), followed by Firmicutes (19.70%) and Bacteroidetes (6.77%) (Fig. 2C) (Additional file 1: Table S1). The main bacterial genus in the HCC tissue group was *Lactobacillus* (14.42%), followed by *Mycoplasma* (9.44%) and *Escherichia* (8.87%). In the normal liver group, the primary genera were *Pseudomonas* (46.05%), *Streptococcus* (8.94%), and *Mucispirillum* (6.50%) (Additional file 1: Fig. S2A) (Additional file 1: Table S2). At the species level, *Mycoplasma*_sp_HU2014, *Escherichia coli*, and *Allobacillus* sp SKP4-8 were the top 3 most abundant taxa in the tumor group, and *Pseudomonas koreensis*, *Streptococcus mutans*, and *Mucispirillum schaedleri* were the richest taxa in the normal group (Additional file 1: Fig. S2B) (Additional file 1: Table S3).

### Differential abundances in bacterial taxa between HCC tissues and normal liver in mice

Statistical analysis was carried out at the levels of phylum, genus and species, which showed significant differences in the abundance of dominant bacterial taxa between the two groups. Compared to normal liver tissues, phylum Firmicutes was markedly increased (P < 0.05), while Proteobacteria was decreased in HCC tissues (P < 0.05, Fig. 2D). At the genus level, 8 genera, including Helicobacter, Muribaculum, and Cutibacterium were remarkable increased, while 2 genera, *Pseudomonas* and *Sphingomonas* were decreased in HCC tissues versus normal liver tissues (all P < 0.05, Fig. 2E). At the species level, *Pseudomonas koreensis* and *Ralstonia* sp UNC404CL21Col showed the most obvious significant differences in the two groups (Fig. 2F).

**Fig. 3:**
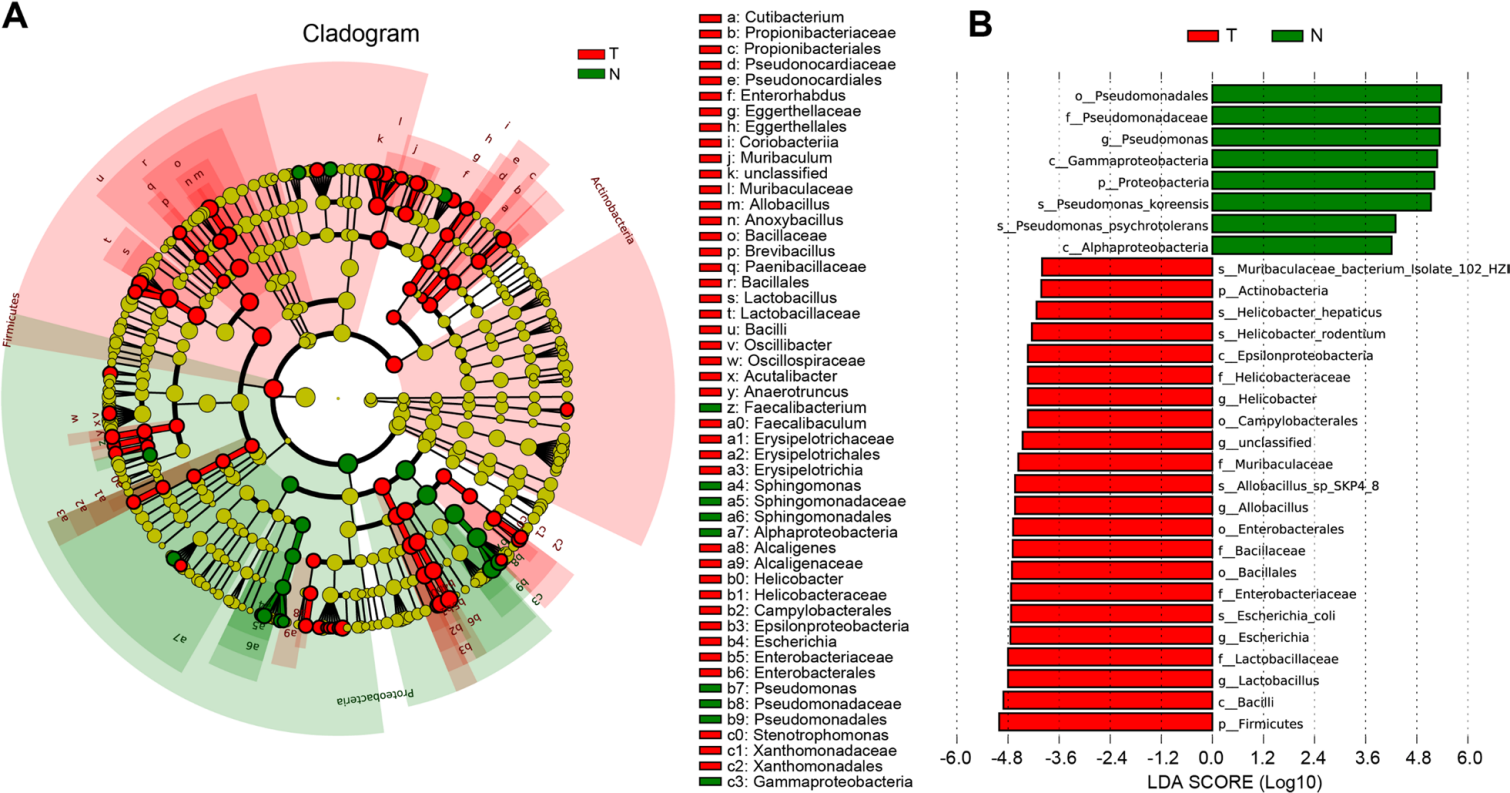
The specific characterization of microbiota between two groups based on LEfSe and LDA analysis. **A** Cladogram generated by the LEfSe represents the taxonomic hierarchical structure of the identified microbial populations. Red nodes and green nodes represent relatively high abundance of species with significant difference in tumor and normal group, respectively. Yellow nodes indicate that there was no significant difference in the comparison of species in the two groups. **B** The histogram of LDA scores showed g significant difference in microbe type and abundance between tumor and normal group. *LEfSe* linear discriminant analysis effect size, *LDA* linear discriminant analysis

Additionally, to discover high-dimensional biomarkers, Linear discriminant analysis (LDA) Effect Size (LEfSe) and LDA score was applied to compare bacterial taxa of the two groups. A cladogram representative of the phylogenetic distribution and their predominant bacteria showed great difference in taxa between HCC tissues and normal liver tissues (Fig. 3A). LDA score displayed the prominent bacteria difference between two groups, which showed that *Pseudomonas* was the most featured taxa in the normal liver group and Firmicutes was the most characteristic taxa in the tumor group (Fig. 3A, B).

### Analysis of the functional differences of microbial genes between the HCC tissues and normal tissues of mice

**Fig. 4:**
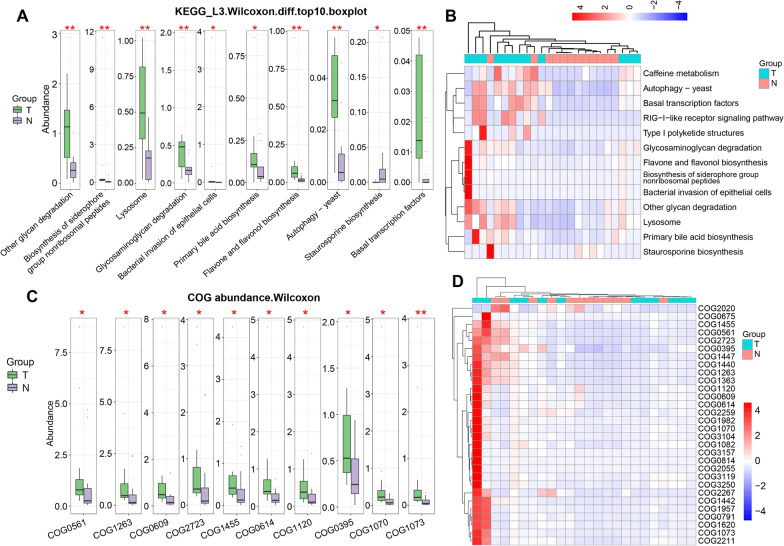
Microbial gene functional enrichment analysis between the two groups. **A** Boxplot of KEGG functional analysis. **B** The clustering heatmap of KEGG analysis. **C** Boxplot of COG analysis. **D** Clustering heatmap of COG analysis

To explore the difference in microbial gene function, we used Kyoto Encyclopedia of Genes and Genomes (KEGG) and Clusters of Orthologous Groups of proteins (COG) functional analysis to determine the top 10 biological pathways and protein differences. In HCC tissues, the microbial gene’s functions were markedly enriched in basal transcription factors, followed by flavone and flavonol biosynthesis and autophagy yeast (Fig. 4A, B). Through COG analysis, we found that several COG proteins were different between the two groups. For example, the level of COG0395 (ABC-type glycerol-3-phosphate transport) in HCC tissues was obviously higher than in normal liver tissues (Fig. 4C, D). Collectively, all the results revealed significant differences in microbial taxa between cancerous and adjacent tissues, suggesting HCC tissues and normal tissues have different microbial environments.

### Metabolites associated with HCC-related microbiota in HCC tissues and normal liver tissues from a mouse model

**Fig. 5:**
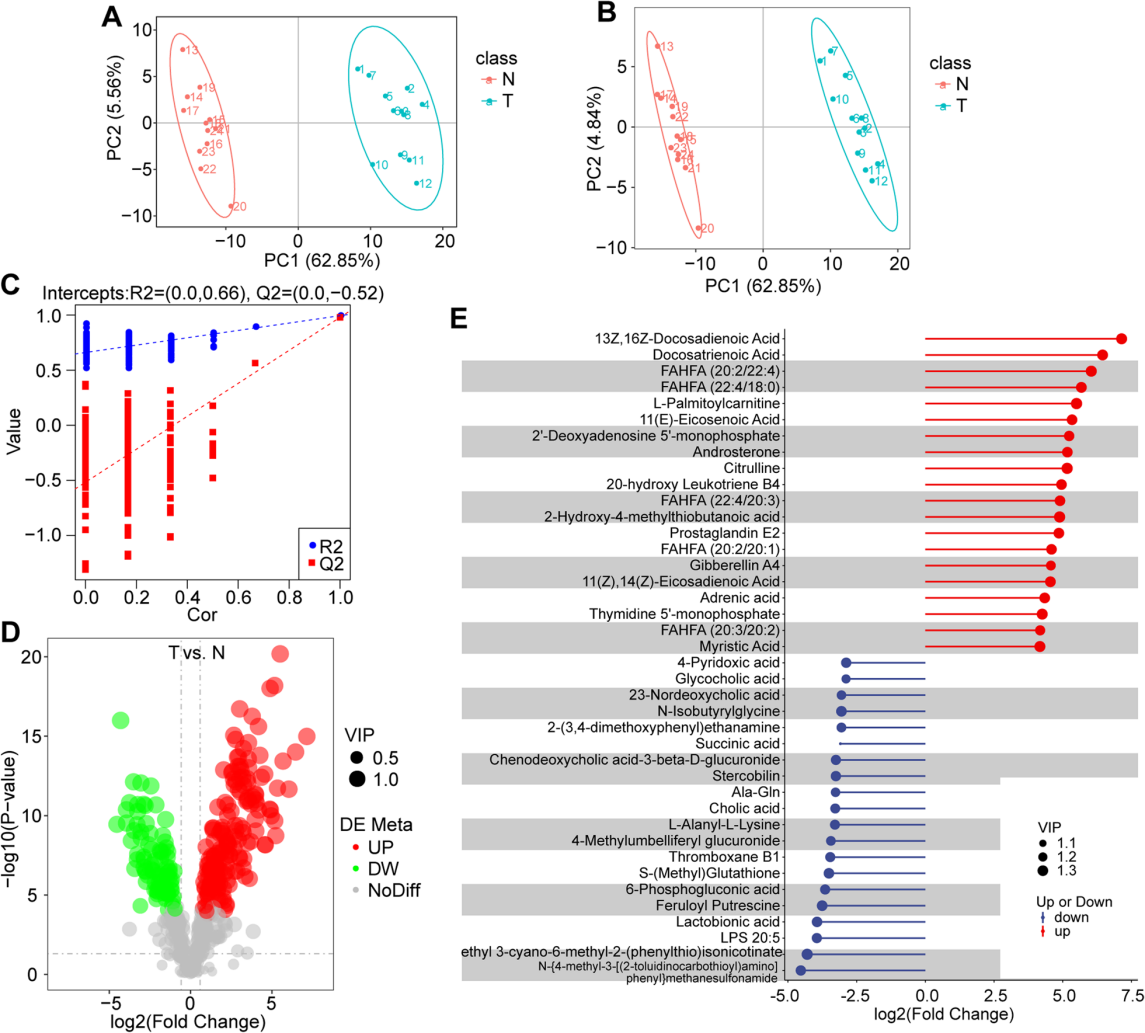
Comparison of the metabolome between HCC tissues and normal tissues. **A** Overall distribution trends of samples between the two groups by principal component analysis (PCA) and partial least squares discrimination analysis (PLS-DA). **B** Different colored points represent samples from different experimental groups, and ellipses represent 95% confidence intervals. **C** PLS-DA model validation based on sevenfold cross-validation and 200 permutation tests. **D** The overall distribution of differential metabolites. **E** Matchstick plots among differential metabolites

To better explore the role of intratumoral bacteria in metabolic homeostasis, we used LC–MS to identify the protein components in HCC tissues and normal liver tissues in a mouse model. Principal component analysis (PCA) and partial least squares discrimination analysis (PLS-DA) methods were applied to observe the overall distribution trend of metabolites, which exhibited noteworthy differences between the two groups (Fig. 5A, B). The model was verified by sevenfold cross-validation and 200 response permutation tests. Variable importance in the projection (VIP) of the PLS-DA model was applied to analyze differentially abundant metabolites, and the reliability of the model is shown in Fig. 5C. As shown in Fig. 5D, the number of total significant metabolites was 258, and 170 significantly upregulated and downregulated 88 metabolites were selected. The top 20 upregulated and downregulated metabolites are shown in Fig. 5E.

**Fig. 6:**
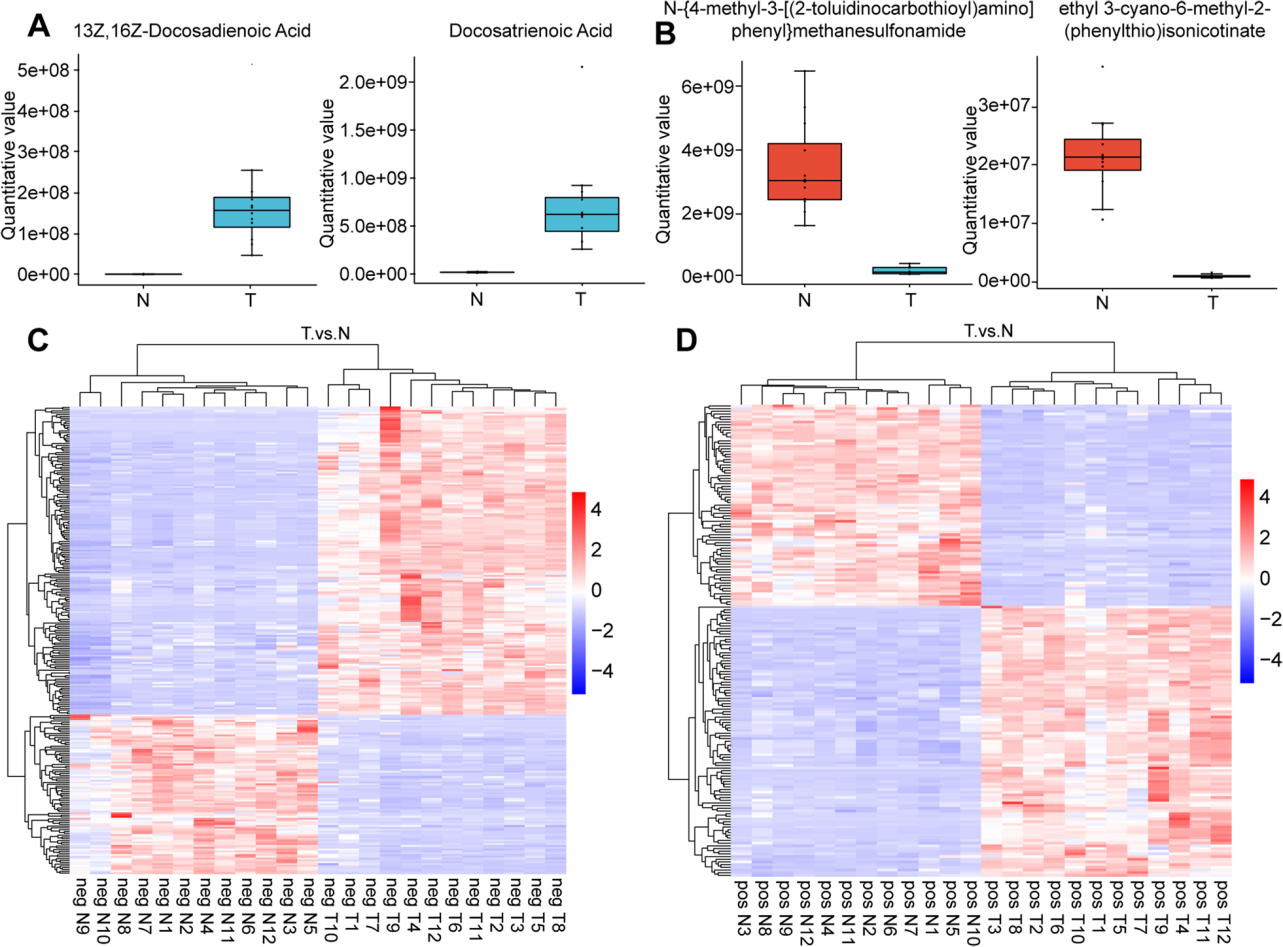
The different metabolites between HCC tissues and normal liver tissues. **A**–**B** Boxplot of four differential metabolites. **C**–**D** Heatmap visualizing metabolites between tumor and normal tissues based on hierarchical clustering analysis

The top 2 upregulated and downregulated metabolites between the two groups, as shown in Fig. 6A, B, were 13Z, 16Z-docosadienoic acid and docosatrienoic acid, which were upregulated in HCC tissues compared with normal liver tissues. N-{4-Methyl-3-[(2-toluidinocarbothioyl)amino]phenyl}methanesulf-onamide and ethyl 3-cyano-6-methyl-2-(phenylthio)isonicotinate were the top 2 downregulated metabolites. To further explore the differences in metabolic patterns of metabolites between HCC tissues and normal liver tissues, we performed cluster analysis of differential metabolites. In negative ionization mode, the different metabolites were clustered, as shown in Fig. 6C. In positive ionization mode, the different metabolites were clustered, as shown in Fig. 6D.

### Correlation analysis of differential metabolites

We analyzed correlations of differential metabolites between HCC tissues and normal liver tissues, with red representing positive correlations and blue dots representing negative correlations. We found that many metabolites were related with each other; for example, D-(-)-glutamine was significantly positively associated with DL-glutamine, α-lactose, and 1-nitrosopyrrolidine (Additional file 1: Fig. S3A), and 13Z,16Z-docosadienoic acid and docosatrienoic acid were negatively correlated with ethyl3-cyano-6-methyl-2-(phenylthio)isonicotinate and positively correlated with L-palmitoycamitine (Additional file 1: Fig. S3B). To clearly demonstrate the association among metabolites, we drew spin diagrams based on the correlation coefficient among the top 20 different metabolites in positive/negative ionization mode (Additional file 1: Fig. S3C, D).

### Functional enrichment analysis of differential metabolites

**Fig. 7:**
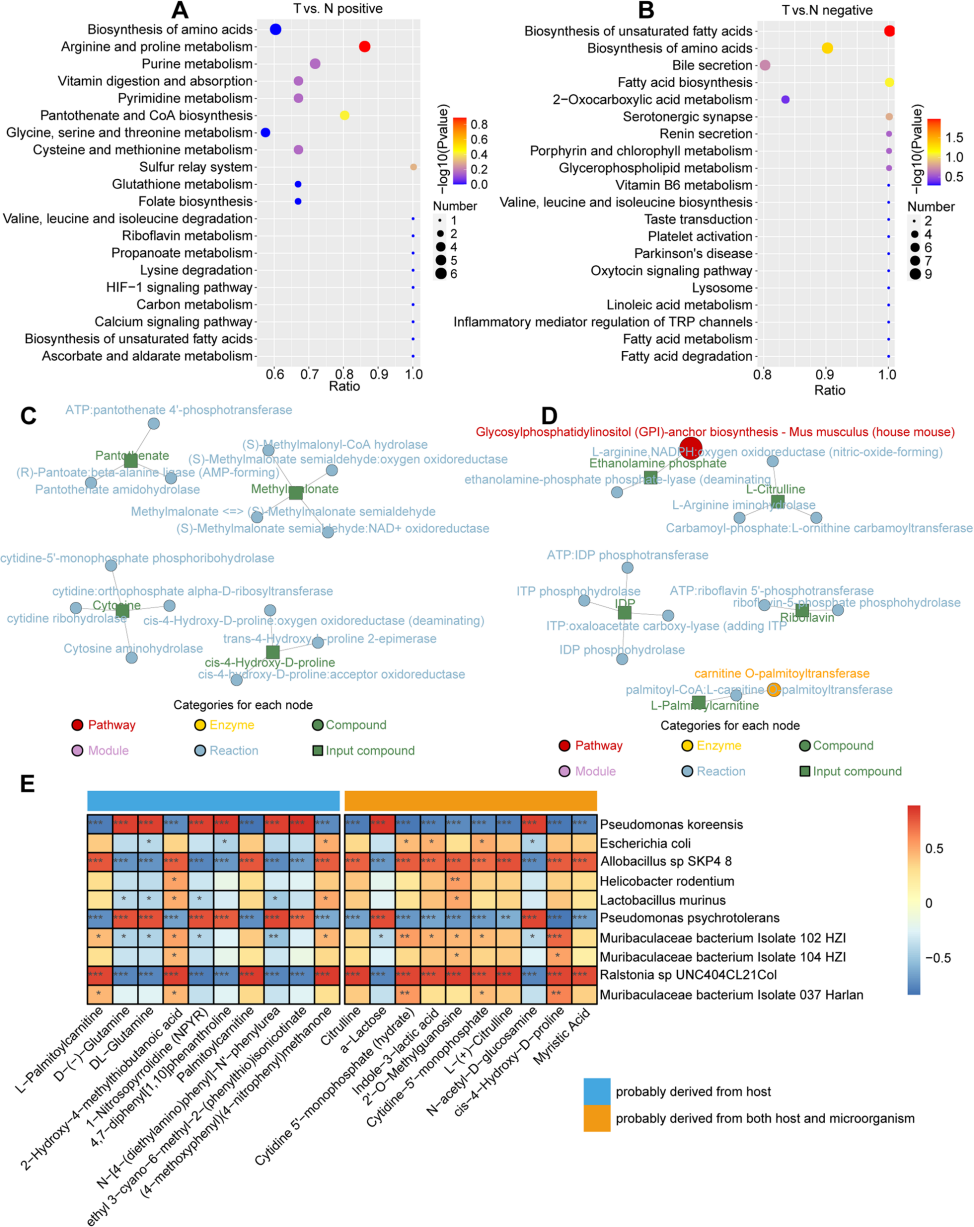
Functional enrichment analysis of differential metabolites. **A**–**B** KEGG enrichment bubble diagram based on positive and negative charges of different metabolites. **C**–**D** Diagram of the KEGG regulatory network based on positive and negative charges of different metabolites. **E** Correlation analysis between top 20 differential metabolites and the top 10 bacterial taxa

We performed KEGG pathway analyses to further understand the biochemical metabolic pathways and signal transduction pathways that the differential metabolites were enriched in. Based on positive and negative ionization modes, we found that the differential metabolites were mainly involved in biosynthesis of amino acids, arginine and proline metabolism, purine metabolism, pyrimidine metabolism, biosynthesis of unsaturated fatty acids, fatty acid biosynthesis, and 2-oxocarboxylic acid metabolism (Fig. 7A, B). Biosynthesis of unsaturated fatty acids and biosynthesis of amino acids were common pathways at the level of positive and negative charges. Furthermore, 4 differential metabolites were selected at the level of positive charge: pantothenate, methylmalonate, cytosine and cis-4-hydroxy-D-proline. At the level of negative charge, a pathway known as glycosylphosphatidylinositol (GPI)-anchor biosynthesis—*Mus musculus* (house mouse) and 5 differential metabolites (ethanolamine phosphate, L-citrulline, IDP, riboflavin and L-palmitoylcarnitine) were identified (Fig. 7C, D).

### Correlation analysis between differential metabolites and intratumoral bacteria

Based on the possible sources of top 20 differential metabolites, we classified them into two categories: metabolites probably derived from the host, and metabolites probably originating from both the host and microorganisms. Subsequently, spearman rank correlation analysis was performed to investigate the association between the top 20 differential metabolites and the top 10 differential intratumoral bacteria (Fig. 7E). The results indicated that the bacteria *Allobacillus sp SKP4 8* and *Ralstonia sp UNC404CL21Col* showed significant positive correlations with most metabolites possibly originating from both the host and microorganisms, including citrulline, cytidine 5'-monophosphate (hydrate), indole-3-lactic acid, 2'-O-methylguanosine, cytidine-5'-monophosphate, L-( +)-Citrulline, cis-4-Hydroxy-D-proline, and myristic Acid, while showed significant negative correlations with a-Lactose and N-acetyl-D-glucosamine. However, the association trend of *Pseudomonas koreensis* and *Pseudomonas psychrotolerans* with metabolites was opposite to *Allobacillus sp SKP4 8* and *Ralstonia sp UNC404CL21Col*. Additionally, we found that downregulated microbes in HCC tissues were significantly positively correlated with some metabolites that were also decreased in HCC tissues. These results suggest that some microbes that are decreased in HCC tumor tissues might be protective microbes that are also involved in the synthetic and metabolic processes of some protective compound metabolites [29].

## Discussion

With the development of intratumor microbiome assessment techniques, more and more intratumor microbiome characteristics have been identified in different types of tumors [8, 30, 31]. In 2020, Nejman et al. detected intratumoral bacteria in seven cancer types and indicated the presence of intratumor bacteria in tumors and immune cells. In addition, the intratumor bacteria were significantly different among the cancer types [13]. Similar to these results, a negative relationship between the intratumoral bacterial load and lymphocyte infiltration was observed in nasopharyngeal carcinoma. Furthermore, the intratumoral bacterial load was associated with the survival rate, including disease-free survival, overall survival and distant metastasis-free survival [32]. A recent study showed that intratumor bacteria could markedly inhibit lung metastasis in a breast tumor mouse model [33]. Taken together, intratumor bacteria could play essential roles in tumor initiation and development, and the potential function and targeted therapy for intratumor bacteria is worth exploring.

Increasing evidence showed that the intratumoral microbiota could play essential roles in HCC progression. Xue et al. assessed the characteristics of bacteria in 47 paired HCC and liver tissues using 16S rRNA sequencing and explored the potential association among differentially expressed genes and metabolites [14]. Based on the analysis of normal liver, peritumoral, and HCC tissue samples, the features of diversity, structure, and abundance were described, and a prognostic prediction model was built in the HCC cohort [12]. In this study, we adopted 2bRAD-M and LC–MS tools to determine the potential association between the intratumoral microbiota and metabolites in mice. Our findings showed that the intratumoral microbiota and metabolites were evidently different in HCC and paired nontumor tissues. To sum up, the features of the intratumoral microbiota might represent potential strategies for HCC diagnosis and treatment, and the intratumoral microbiota could provide clues for therapeutic decision-making in HCC.

Studies have shown significant differences between cancer and normal tissues, including between genes, metabolites, and RNA methyladenosine [34–37]. Our research classified the Top20 differential metabolites based on their potential sources into two categories: probably derived from the host, and probably derived from both the host and microorganism. Furthermore, we evaluated the relationship between the tumor microbiota and metabolites in a liver cancer mouse model. The results indicated a significant positive correlation between the metabolites D-( −)-Glutamine and DL-Glutamine, probably derived from the host, and Pseudomonas koreensis and Pseudomonas psychrotolerans. It suggests that the microbiota may indirectly influence the abundance of metabolites by affecting the host's metabolic status. In recent years, the role of glutamine metabolism imbalance in the pathogenesis of HCC has been increasingly emphasized [37, 38]. Functionally, decreased GOT2 expression has been found to promote glutaminolysis through glutamine metabolism and contribute to HCC progression [38]. In addition, Wei et al. found that HMGB1 regulated glutamine metabolism in HCC cell through dual mechanisms. On the one hand, HMGB1 could promote glutamine synthetase expression via the mTORC2-AKT-C-MYC pathway. On the other hand, HMGB1 could inhibit glutamate dehydrogenase by inducing the mTORC1 pathway to down-regulate SIRT4 [39]. These findings highlight the importance of studying tumor metabolism as biomarkers for HCC diagnosis and prognosis prediction.

Accumulated evidence suggests that there is a close relationship between intratumoral bacteria and metabolite [40, 41]. Microbiota derived metabolites play a role in regulating the tumor microenvironment [42]. Citrulline, for example, is a metabolite that derived from both host and microbial sources [43, 44]. Citrulline is known to have two synthetic pathways that are primarily completed in the small intestine [45]. In our study, citrulline has strong correlation with *muribaculaceae bacterium isolate 102 HZI*, *Ralstonia sp UNC404CL21Col* and *Allobacillus sp SKP48*, suggesting that these bacteria in tumors may be involved in the metabolic pathway of citrulline in the liver. Although the evidence for the association between the microbiota and metabolites is relatively weak, it provides some clues for us to further explore the specific function and mechanism of metabolites derived from intratumoral bacteria.

In conclusion, we utilized 2bRAD-M and LC–MS to describe the features of bacteria and metabolites in an HCC mouse model. Our findings might provide clues for further study of the potential function of bacteria and metabolites in tumor tissues.

## Conclusion

The intratumoral microbial signature of the microbiome in HCC tissues and paired normal tissues was demonstrated. The tumor microenvironment can be better understood by the correlation between microbial species and metabolites. Differential bacteria and its metabolites might be new biomarkers for prognosis and treatment of HCC patients.

### Supplementary Information

**Additional file 1: Figure S1.** Bacterial abundance and distribution in HCC tissues and normal liver tissues from a mouse model. The barplot and heatmap of the top 15 species in abundance are provided based on the taxonomic levels of **(A)** phylum, **(B)** genus, and **(C)** species. **Figure S2.** Composition and relative abundance of microbiota in HCC tissues versus normal liver tissues. (A) The composition and relative abundance of microbiota at genus level. (B) The composition and relative abundance of microbiota at species level. **Figure S3.** Correlation analysis of differential metabolites. (**A-B)** Correlation analysis of differential metabolites based on positive and negative charges. (**C-D**) Chord chart showing the correlation analysis of differential metabolites based on positive and negative charges. The color of the dot represents different metabolites, the size of the dot represents the size of the log2 fold change, the thickness of the line between metabolites represents the correlation between two metabolites, the blue line represents the negative correlation, and the red line represents the positive correlation. **Table S1.** The main composition and relative abundance of microbiota at phylum level in HCC and normal liver tissues. **Table S2.** The main composition and relative abundance of microbiota at genus level in HCC and normal liver tissues. **Table S3.** The main composition and relative abundance of microbiota at species level in HCC and normal liver tissues.

## Data Availability

The datasets used and/or analyzed during the current study are available from the corresponding author upon reasonable request.

## Abbreviations

HCC: Hepatocellular carcinoma
LPS: Lipopolysaccharide
2bRAD-M: 2bRAD sequencing for microbiome
FISH: Fluorescence in situ hybridization
H&E: Hematoxylin and eosin
IHC: Immunohistochemistry
PBS: Phosphate buffer solution
LC–MS: Liquid chromatography-mass spectrometry
PCA: Principal component analysis
PLS-DA: Partial least squares discriminant analysis
HCA: Hierarchical clustering
LDA: Linear discriminant analysis
KEGG: Kyoto Encyclopedia of Genes and Genomes
COG: Clusters of orthologous groups
VIP: Variable importance in the projection
LEfSe: Linear discriminant analysis effect size
