# Supplementary material for: The interaction between intratumoral bacteria and metabolic distortion in hepatocellular carcinoma
Source: J Transl Med. 2024 Mar 4;22:237. doi: 10.1186/s12967-024-05036-7 (PMC10910819; doi:10.1186/s12967-024-05036-7)
Supplement: Supplementary file 1 — Additional file 1: Figure S1. Bacterial abundance and distribution in HCC tissues and normal liver tissues from a mouse model. The barplot and heatmap of the top 15 species in abundance are provided based on the taxonomic levels of (A) phylum, (B) genus, and (C) species. Figure S2. Composition and relative abundance of microbiota in HCC tissues versus normal liver tissues. (A) The composition and relative abundance of microbiota at genus level. (B) The composition and relative abundance of microbiota at species level. Figure S3. Correlation analysis of differential metabolites. (A-B) Correlation analysis of differential metabolites based on positive and negative charges. (C-D) Chord chart showing the correlation analysis of differential metabolites based on positive and negative charges. The color of the dot represents different metabolites, the size of the dot represents the size of the log2 fold change, the thickness of the line between metabolites represents the correlation between two metabolites, the blue line represents the negative correlation, and the red line represents the positive correlation. Table S1. The main composition and relative abundance of microbiota at phylum level in HCC and normal liver tissues. Table S2. The main composition and relative abundance of microbiota at genus level in HCC and normal liver tissues. Table S3. The main composition and relative abundance of microbiota at species level in HCC and normal liver tissues. [file 12967_2024_5036_MOESM1_ESM.pdf]

Supplemental file for

The interaction between intratumoral bacteria and metabolic distortion in hepatocellular carcinoma

Chen Xue[a†], Xinyu Gu[b†], Qingmiao Shi[a†], Xiao Ma[a], Junjun Jia[c], Yuanshuai Su[a], Zhengyi Bao[a], Juan Lu[a*], Lanjuan Li[a*]

[a] State Key Laboratory for Diagnosis and Treatment of Infectious Diseases, National Clinical Research Center for Infectious Diseases, National Medical Center for Infectious Diseases, Collaborative Innovation Center for Diagnosis and Treatment of Infectious Diseases, The First Affiliated Hospital, Zhejiang University School of Medicine, Hangzhou, Zhejiang, China.

[b] Department of Oncology, The First Affiliated Hospital, College of Clinical Medicine, Henan University of Science and Technology, Luoyang, Henan, China.

[c] Division of Hepatobiliary and Pancreatic Surgery, Department of Surgery, The First Affiliated Hospital, Zhejiang University School of Medicine, Hangzhou, Zhejiang, China.

[†] These authors contributed equally to this work: Chen Xue, Xinyu Gu, and Qingmiao Shi.

***Corresponding author**

Lanjuan Li & Juan Lu,

State Key Laboratory for Diagnosis and Treatment of Infectious Diseases, The First Affiliated Hospital, Zhejiang University School of Medicine, Hangzhou, Zhejiang, China. Tel: 86 0571-87236459; Email: ljli@zju.edu.cn (Lanjuan Li), lujuanzju@zju.edu.cn (Juan Lu).

This file includes:

Supplementary Figures 1 to 3.

Supplementary Tables 1 to 3.

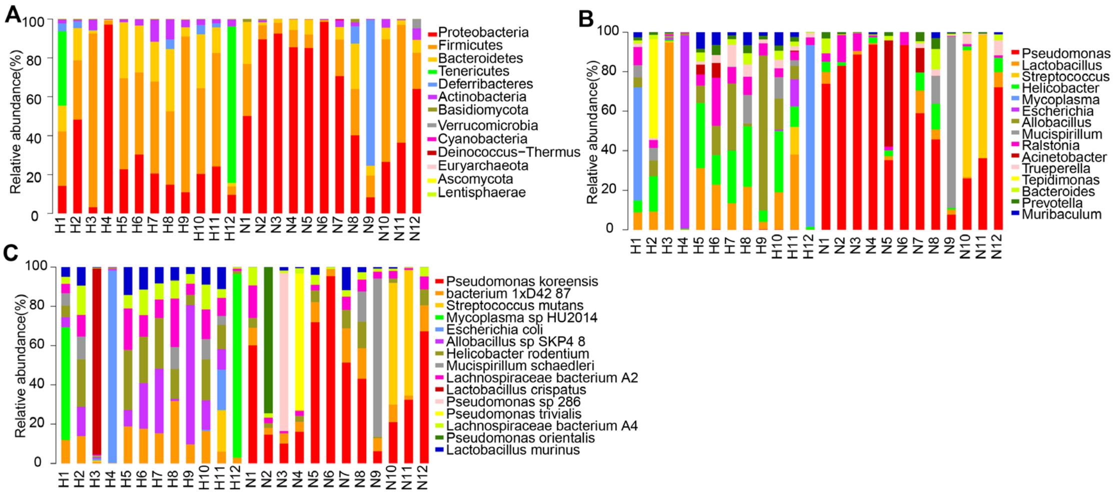

Figure S1. Bacterial abundance and distribution in HCC tissues and normal liver tissues from a mouse model. The barplot and heatmap of the top 15 species in abundance are provided based on the taxonomic levels of **(A)** phylum, **(B)** genus, and **(C)** species.

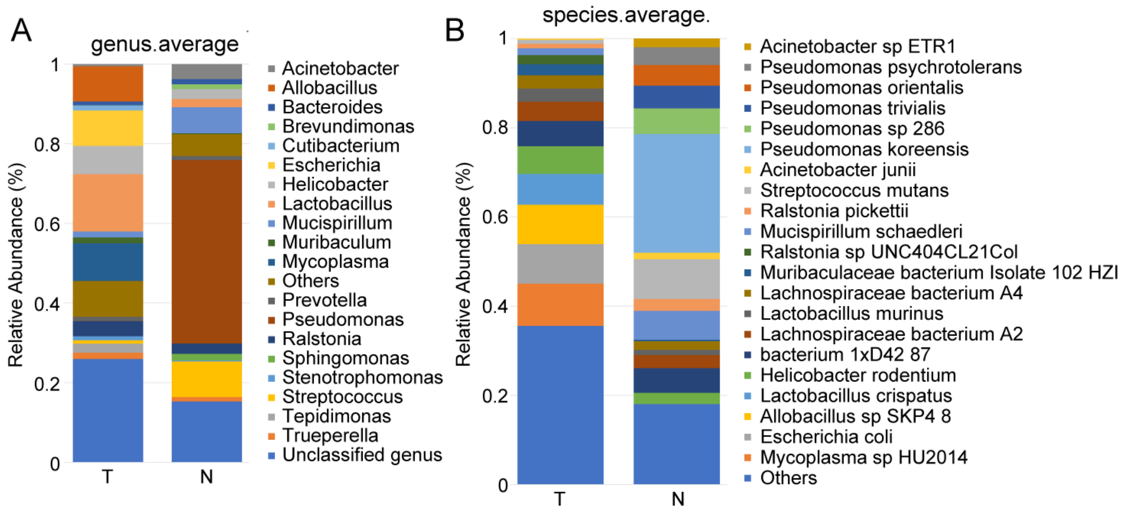

Figure S2. Composition and relative abundance of microbiota in HCC tissues versus normal liver tissues. (A) The composition and relative abundance of microbiota at genus level. (B) The composition and relative abundance of microbiota at species level.

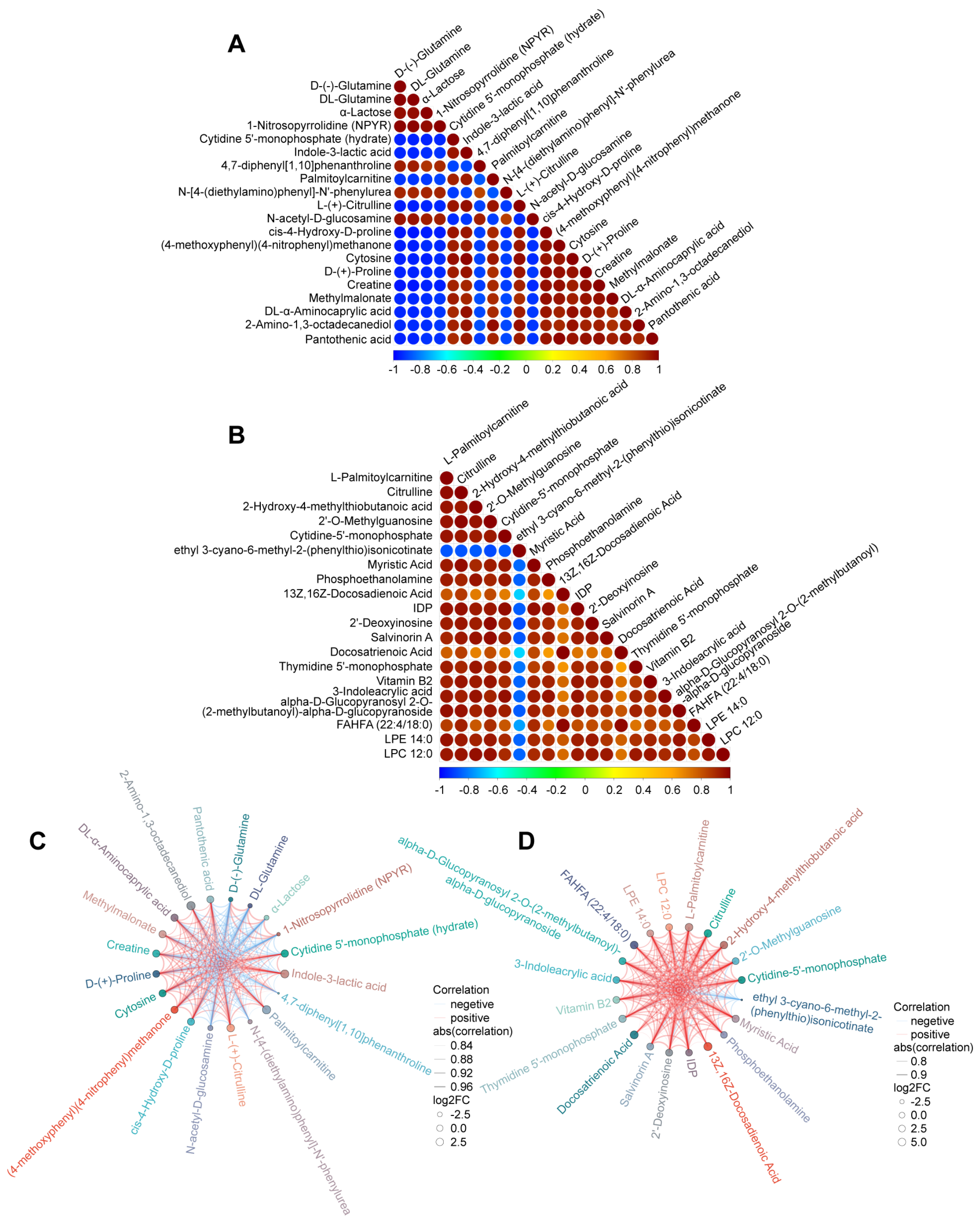

Figure S3. Correlation analysis of differential metabolites. (**A-B)** Correlation analysis of differential metabolites based on positive and negative charges. (**C-D**) Chord chart showing the correlation analysis of differential metabolites based on positive and negative charges. The color of the dot represents different metabolites, the size of the dot represents the size of the log2 fold change, the thickness of the line between metabolites represents the correlation between two metabolites, the blue line represents the negative correlation, and the red line represents the positive correlation.

Table S1. The main composition and relative abundance of microbiota at phylum level in HCC and normal liver tissues

phylum	HCC tissues	normal liver tissues	P	FDR_P
Firmicutes	0.395858	0.196971	0.040676	0.186699
Proteobacteria	0.249124	0.586587	0.005515	0.077209
Bacteroidetes	0.14002	0.067742	0.053343	0.186699
Tenericutes	0.094413	0	0.185106	0.450324
Unclassified_phylum	0.068173	0.062844	0.723881	0.723881
Actinobacteria	0.03499	0.013702	0.052952	0.186699
Deferribacteres	0.01508	0.064962	0.401332	0.468221
Others	0.002342	7.19E-03		

Table S2. The main composition and relative abundance of microbiota at genus level in HCC and normal liver tissues

genus	HCC tissues	normal liver tissues	P	FDR_P
Unclassified_genus	0.259563	0.152818	0.071313	0.398836
Trueperella	0.015913	0.011319	0.504598	0.546648
Tepidimonas	0.022314	0	0.325356	0.398836
Streptococcus	0.008737	0.08938	0.20008	0.398836
Stenotrophomonas	0.010275	0.002346	0.033318	0.374519
Sphingomonas	7.70E-05	0.016565	6.04E-05	0.003141
Ralstonia	0.037837	0.02623	0.453024	0.501218
Pseudomonas	0.000523	0.460531	4.43E-06	0.000461
Prevotella	0.010342	0.010288	0.989154	0.989154
Others	0.09	0.05451		
Mycoplasma	0.094413	0	0.185106	0.398836
Muribaculum	0.014605	0.002711	0.009749	0.170644
Mucispirillum	0.01508	0.064962	0.401332	0.458666
Lactobacillus	0.144187	0.020955	0.083063	0.398836
Helicobacter	0.071194	0.024908	0.009845	0.170644
Escherichia	0.088782	0	0.275542	0.398836
Cutibacterium	0.012182	0	0.028464	0.370038
Brevundimonas	0.000774	0.011827	0.286545	0.398836
Bacteroides	0.009289	0.012991	0.526958	0.55922
Allobacillus	0.088561	0	0.061257	0.398836
Acinetobacter	0.00589	0.037658	0.315088	0.398836

Table S3. The main composition and relative abundance of microbiota at species level in HCC and normal liver tissues

species	HCC tissues	normal liver tissues	P	FDR_P
Others	3.56E-01	0.180823		
Mycoplasma_sp_HU2014	0.094413	0	0.185106	0.3764
Escherichia_coli	0.088782	0	0.275542	0.3764
Allobacillus_sp_SKP4_8	0.088561	0	0.061257	0.3764
Lactobacillus_crispatus	0.069079	0	0.328183	0.3764
Helicobacter_rodentium	0.06183	0.024908	0.025918	0.3764
bacterium_1xD42_87	0.056567	0.055494	0.937083	0.952832
Lachnospiraceae_bacterium_A2	0.043244	0.029465	0.252481	0.3764
Lactobacillus_murinus	0.029902	0.011669	0.050983	0.3764
Lachnospiraceae_bacterium_A4	0.029148	0.019244	0.14294	0.3764
Muribaculaceae_bacterium_Isolate_102_HZI	0.024988	0.003412	0.003	0.120991
Ralstonia_sp_UNC404CL21Col	0.020552	0	0.001059	0.095398
Mucispirillum_schaedleri	0.01508	0.064962	0.401332	0.437489
Ralstonia_pickettii	0.010295	0.02623	0.182312	0.3764
Streptococcus_mutans	0.008737	0.08938	0.20008	0.3764
Acinetobacter_junii	0.002505	0.01462	0.246308	0.3764
Pseudomonas_koreensis	0	0.266033	7.51E-05	0.018171
Pseudomonas_sp_286	0	0.057293	0.328183	0.3764
Pseudomonas_trivialis	0	0.051155	0.328183	0.3764
Pseudomonas_orientalis	0	0.046349	0.328183	0.3764
Pseudomonas_psychrotolerans	0	0.0397	0.001812	0.109621
Acinetobacter_sp_ETR1	0	0.019262	0.328183	0.3764
